# Stem cells in homeostasis and cancer of the gut

**DOI:** 10.1186/s12943-019-0962-x

**Published:** 2019-03-30

**Authors:** Maartje van der Heijden, Louis Vermeulen

**Affiliations:** Amsterdam UMC, University of Amsterdam, Laboratory for Experimental Oncology and Radiobiology, Center for Experimental and Molecular Medicine, Cancer Center Amsterdam and Amsterdam Gastroenterology and Metabolism, Meibergdreef 9, 1105 Amsterdam, AZ Netherlands

**Keywords:** Intestinal stem cells, Cell plasticity, Cancer stem cells, Tumor (micro-)environment, Colorectal cancer

## Abstract

The intestinal epithelial lining is one of the most rapidly renewing cell populations in the body. As a result, the gut has been an attractive model to resolve key mechanisms in epithelial homeostasis. In particular the role of intestinal stem cells (ISCs) in the renewal process has been intensely studied. Interestingly, as opposed to the traditional stem cell theory, the ISC is not a static population but displays significant plasticity and in situations of tissue regeneration more differentiated cells can revert back to a stem cell state upon exposure to extracellular signals. Importantly, normal intestinal homeostasis provides important insight into mechanisms that drive colorectal cancer (CRC) development and growth. Specifically, the dynamics of cancer stem cells bear important resemblance to ISC functionality. In this review we present an overview of the current knowledge on ISCs in homeostasis and their role in malignant transformation. Also, we discuss the existence of stem cells in intestinal adenomas and CRC and how these cells contribute to (pre-)malignant growth. Furthermore, we will focus on new paradigms in the field of dynamical cellular hierarchies in CRC and the intimate relationship between tumor cells and their niche.

## Background

The intestinal tract is a widely studied organ with a multitude of functions. Besides its primary purpose to absorb nutrients and remove feces, it is also a major player in the regulation of metabolic and immune processes in the human body. These different functions reflect the complexity of this organ and highlight the enormous interplay that exists between the extensive cellular and non-cellular parts that make up the intestinal tract including: epithelial cells, immune cells, stromal cells, hormones and neurotransmitters, nutrients, the microbiome and many more. Hence, it comes as no surprise that many diseases are associated with malfunctioning of the intestine, such as infectious and autoimmune disorders. Colorectal cancer (CRC) is another common disease that arises from the colonic epithelial layer.

CRC is a significant cause of cancer related death and worldwide the incidence is still increasing [1]. Early stage disease is often still curable but the availability of effective curative therapies for disseminated CRC is very limited. Throughout the years much emphasis has been put on genetic causes of cancer, in particular the oncogenic driver and tumor-suppressor gene mutations [2]. For CRC, already decades ago, genomic aberrations that are associated with the progression of polyps and adenomas to CRC were identified [3]. To date CRC is the prime example of step-wise carcinogenesis. However, the biology of CRC contains so many more facets than the genetic aberrancies present within tumor cells. In particular the (micro-)environment is of great relevance in shaping the clinical presentation of the disease, and key to understanding process including metastasis formation and therapy failure [4–6]. As for other cancer types, another phenomenon that is hugely complicating therapy responses is the observed inter- and intratumor heterogeneity [7, 8]. First of all, *inter*tumor heterogeneity referring to the differences between patients presenting with CRC, is extensive, and relates to clinical as well as genetic properties. Transcriptomic profiling of CRCs led to the identification of four main CRC subtypes [9, 10]. These subtypes differ in genetic aberrations, composition of the immune infiltrate and other features of the stromal compartment, as well as the clinical outcomes of the disease. Underneath these molecular subgroups lies another important layer of complexity, namely the cellular *intra*tumor heterogeneity. It has been recognized that CRCs contain extensive genetic variability reflecting the ongoing accumulation of mutations and competition for space and nutrients. Simultaneously, individual CRCs contain cells with different differentiation grades, also in genetically homogenous clones. It is thought that these cells reflect different stages of differentiation that mirror the differentiation patterns found in the normal intestine. It has also been postulated that these various degrees of differentiation are accompanied by functional differences, with stem cell-like cells: cancer stem cells (CSCs) driving tumor growth and progression. This hierarchical tumor model has also served as an attractive explanation for therapy failure as it has been described that CSCs are more resistant to conventional therapies and therefore are likely seeds of tumor relapse.

Stem cell biology plays an equally important role in another aspect of CRC biology. It is believed that intestinal stem cells (ISCs) are the cell of origin of the large majority of CRCs [11]. Therefore, understanding the properties of ISCs in detail is likely to contribute to a better understanding of CRC development and progression. In the past decade, major advances have been made to reveal the ISC identity (Table1). Crucially, it appears that the ISC state and consequently the ISC identity is highly dynamic. Accordingly, pinpointing one demarcated group of cells as the ISCs has been proven difficult. Similarly, the plasticity of ISCs is also reflected in CSCs as recent work demonstrates, and prone to greatly hamper the efficacy of CSC-specific targeted therapies. In this review, we provide an overview of ISCs in homeostasis and tumor initiation, and crucially their interplay with the environment which directly impacts on cellular differentiation grades. In analogy with this, we will review the current knowledge on colorectal CSC biology. As opposed to the initially rigid CSC theory, that viewed CSCs as rare and intrinsically distinct entities, it becomes increasingly evident that the CSC state is subjected to cellular plasticity and importantly, might be much more common than previously expected. To conclude, we will highlight the current insights on how stem cell features potentially impede the effects of anti-cancer therapy in CRC.

### Intestinal stem cells

#### Intestinal stem cells in homeostasis

**Fig. 1:**
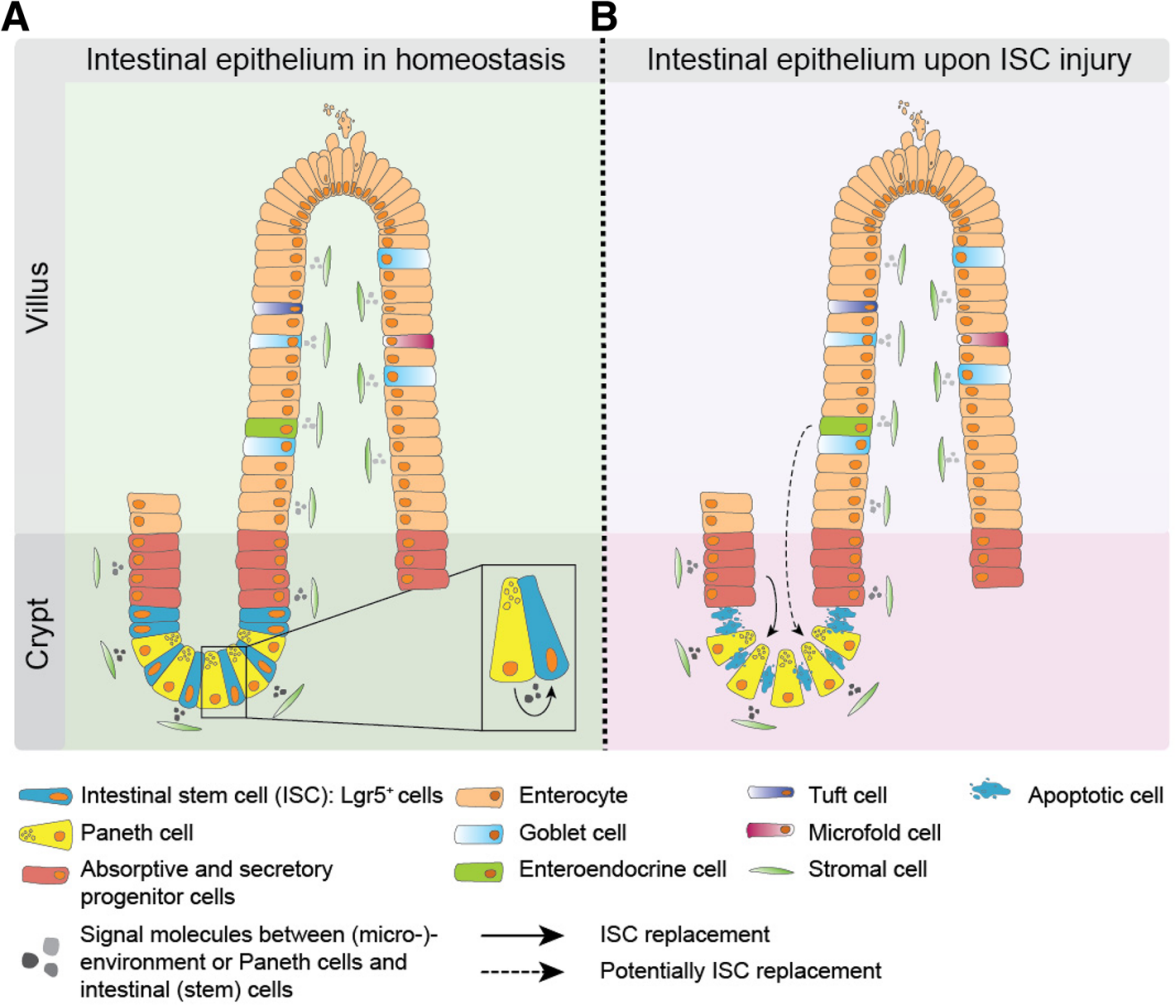
The intestinal epithelium. (**a**) The intestinal lining consists of an epithelial monolayer covering invaginations (crypts) and finger like protrusions (villi, only in the small intestine). Intestinal stem cells (ISC) reside in the bottom of the crypts, absorptive and secretory progenitor cells directly above the ISC zone, and more differentiated cells towards the top of crypts and on the villi. Intestinal progenitor and differentiated cells move upwards due to the massive tissue renewal fueled by the ISCs. This is a continuous process and it only takes several days before differentiated cells undergo apoptosis and are shed into the gut lumen. (**b**) The ISC compartment is sensitive to cytotoxic injury, such as irradiation. Consequently, upon DNA damage ISCs undergo apoptosis. The progenitor cells located higher up in the crypt replace the loss of ISCs and due to the new topological position regain niche signals, which then install ISC activity. Therefore, the ISC compartment is a dynamic population and progenitor- and potentially fully differentiated cells, show an enormous cellular plasticity upon ISC loss

The intestinal lining consists of a monolayer of epithelial cells covering the stromal compartment of the gut, and is characterized by a tight regulation and an immense turn-over capacity. All intestinal epithelium cells are replaced every 3–4 days in mice and this renewal rate is speculated to be approximately every week in the human colon [12]. This rapid renewal is likely to be important for limiting the amount of damaged epithelial cells due to the many bacteria and (toxic) chemicals that pass by inside the lumen and which are continuously in direct contact with these cells. The small intestinal epithelial layer contains a heterogeneous pool of cells, starting from the bottom of the crypts towards the top of the villi (Fig. 1a). Globally, along the crypt-villus axis, the ISCs reside in the bottom region of the crypt, whereas progenitors and differentiated cells are found more towards the top of crypts and villi, respectively [13]. The most abundant differentiated cell is the absorptive enterocyte. Furthermore, there are various secretory cells, only consisting of a few percent of all cells, which comprises the mucus producing Goblet cells, Paneth cells, Enteroendocrine cells, and the very rare Tuft and Microfold (M) cells [13]. All these cells contribute to specific tasks of the intestine.

**Table 1:** Different characteristics of intestinal (cancer) stem cell behavior

*ISC or CSC phenotype*: this indicates the identification of stem cells based on the expression of certain markers or pathway-activities, which are associated with stem cell features, such as self-renewal and multi-potency.	
*ISC or CSC activity*: this indicates whether a specific intestinal or CRC cell population shows active stem cell behavior as found by clonal lineage tracing experiments.	
*ISC or CSC potential*: this indicates an inactive stem cell state in homeostasis but the ability of cells to reversibly undergo dedifferentiation in specific circumstances. For example, for differentiated cells in an inflammatory environment and CRC cells that receive specific stromal signals, i.e. Osteopontin.	
*ISC or CSC functionality*: this indicates the underlying stem cell dynamics of all active ISCs or CSCs present in a specific situation, e.g. during ISC homeostasis or CRC growth.

The incredible epithelial turnover is sustained by ISCs that reside in the bottom of the crypts. With the development of lineage tracing technology our knowledge of ISCs underwent a transformation. In a seminal study from the laboratory of Hans Clevers leucine-rich-repeat-containing G-protein-coupled receptor 5 expressing (Lgr5^+^) cells were demonstrated to function as bona fide stem cells [14]. These Lgr5^+^ cells, are slender cells squeezed in between the Paneth cells and were already previously described as crypt base columnar cells (CBCs) by Cheng and Leblond [15, 16]. Already in 1974 these CBCs were considered as rare, long-lived and slow-cycling cells [15, 16]. In fact, CBCs are actively cycling and continuously contributing to fuel the whole crypt-villus axis with newly generated epithelial cells. Subsequently, many markers have been identified in lineage tracing experiments of which most directly overlap with the Lgr5^+^ population, for example: B lymphoma Mo-MLV insertion region 1 homolog (*Bmi1*) [17], HOP homeobox (*Hopx*) [18], SPARC related modular calcium binding 2 (*Smoc2*) [19], mouse telomerase reverse transcriptase (m*Tert*) [20], SRY-box 9 (*Sox9*) [21], leucine rich repeats and immunoglobulin like domains 1 (*Lrig1*) [22], and prominin 1 (*Prom1*) [23, 24]. Virtually all these markers are located primarily in the CBC position intermingled with Paneth cells and in position + 4 right above the bottom of the crypt. Paneth cells provide a niche for the ISCs by critically excreting factors that contribute to the ISC state, in particular Wnt ligands [25]. In the bottom of murine crypts, ~14 highly proliferative and equipotent Lgr5^+^ cells are found that divide every day and replace each other in a stochastic fashion, in a process that is referred to as neutral drift [26–28]. In time this leads to niche succession and ultimately the generation of a clonally related population within the crypt [26–28]. Intriguingly, the cellular position is closely linked to the function of intestinal cells as cells from the Lgr5^+^ population do not have equipotent chance in gaining niche fixation [29]. Niche fixation chances are in favor of the bottom stem cells as they are less likely to be replaced, simply due to their topological position close to the niche [29]. By using a marker-free lineage tracing approach in combination with a quantitative stochastic model we demonstrated that only five to seven ISCs are predominantly participating in constantly achieving niche fixation [30]. Interestingly, these ISCs do not act alone but are involved in an intimate relationship with their (micro-)environment as we will describe hereafter.

#### Intestinal cell plasticity

The intestine demonstrates impressive regeneration potential in case of intestinal injury, as depletion of all Lgr5^+^ ISCs does not result in crypt loss and complete regeneration of the affected crypts occurs [31, 32]. Fast-cycling Lgr5^+^ cells are vulnerable to DNA damage caused by for example radiation or cytostatic agents because of their highly proliferative state. In response to loss of Lgr5^+^ cells due to these types of injury, two cell types are believed to be responsible for replenishing the ISC pool and sustaining epithelial homeostasis; 1) slow-cycling, quiescent cells at the + 4 position (also called ‘+ 4’ cells) within crypts and 2) absorptive and secretory progenitors (Fig. 1b) [33–36]. However, it is still debated whether the ‘+ 4’ cells are truly distinct from the Lgr5^+^ cells as these two populations show evident overlap in marker expression, e.g. *Bmi1*, *mTert*, *Lrig1*, *Hopx, Atoh1* and *Mex3A* [35, 37, 38]. Additionally, also the Paneth precursor label-retaining cell (LRC) population on the ‘+ 4’ position can acquire stem cell properties upon tissue injury [39]. Recently it was found that despite differential lineage fates, a subpopulation of Lgr5^+^ cells and LRCs show overlapping transcriptomic signatures, indicating not a clear separation between ‘1–3’ and ‘+ 4’ positioned crypt cells [37]. In conclusion, CBC cells display functional marker expression differences based on their location within the crypt bottom but seem uniformly capable of multipotent behavior, albeit in different circumstances. Two factors seem important for this bidirectional conversion: 1) the intrinsic ability to switch cell fate, e.g. by chromatin remodeling [40], and 2) receiving niche signals for reversibly gaining ISC phenotype and functionality [25]. Crucially, retrieval of specific niche factors, as provided by Paneth cells, due to the newly obtained topological position following CBC loss is necessary to re-gain ISC activity [25]. Also, interestingly, it was found that upon transitioning from ISC to differentiated cell state major changes take place on the chromatin accessibility sites of many cell-type specific genes [40]. When required, these sites can completely revert from a closed to an open state and thereby switching between different cellular functionalities. It is plausible that dynamic chromatin remodeling is one of the key factors underlying the cell-fate switch [40]. In contrast, the epigenetic status as witnessed by genome-wide DNA methylation patterns remains relatively stable upon (de-)differentiation [41, 42]. However, it remains yet unknown whether there is a *point-of-no-return* maturation state for undergoing de-differentiation (Fig. 1b). Recent work has indicated that even terminally differentiated Paneth cells and late-stage entero-endocrine cells, still have the capacity to switch back to an ISC state, indicating that conceivably any intestinal epithelial cell is equipped with this potential [43–45].

#### Signals regulating intestinal stem cells

As in other organ systems, ISCs rely heavily on signals from the stem cell environment, i.e. the niche [46]. The Paneth cells constitute a key part of the ISC niche and are a source of factors like epithelial growth factor (EGF), transforming growth factor-α (TGF-α), Wnt3 and the Notch ligand Delta-like 4 (Dll4) [25]. Wnt pathway activation is arguably the most important pathway for installing the ISC phenotype and seems to overrule other pathways to do so [25, 47]. The mesenchymal cell layer surrounding CBC cells is also an important source of Wnt signals [48–50]. In addition, Notch, EGFR/MAPK and ErbB are other signaling routes, that are important for ISC maintenance [25, 51]. Bone morphogenetic protein (BMP) signaling, on the other hand, inhibits stem cell expansion and is actively repressed by the antagonist Noggin in the niche [52, 53]. BMP and Ephrin-B signaling are indeed increasingly expressed from the crypt bottom towards the villus tips in a transient manner thereby promoting differentiation of epithelial cells when these cells move upwards on the crypt-villus axis [54]. Conversely, inactivation of the BMP pathway results in excessive ISC niche expansion [55]. Similarly, deprivation from Wnt signals due to the cellular position directs cells towards differential lineages [56]. The heterogeneous progenitor compartment is regulated by an interplay of differently expressed pathways [13]. Stochastic processes as well as signals received from stroma or neighboring cells underlie the complex coordination of the formation of various intestinal lineages (lateral inhibition chromatin remodeling) [42]. Instantly after cells leave the Wnt-rich environment signaling routes such as Notch, BMP and EGFR/MAPK come into play. Notch activation in progenitor cells is mediated by paracrine signaling through secretion of Delta-like 1 (Dll1) and Dll4 ligands and leads to an absorptive lineage formation [57]. In agreement, chemical inactivation of Notch signals drives progenitor cells towards the secretory fate [58]. Conversely, it is hypothesized that stochastic Notch repression in progenitor cells induces also *Atoh1* (also known as *Math1*) expression, which is essential for commitment towards the secretory lineage [59]. Furthermore, the difference between active and quiescent ‘+ 4’ Lgr5^+^ cells potentially results from differences in Wnt and EGFR/MAPK activity. The slow-cycling ‘+ 4’ LRCs are Wnt^high^ but have reduced EGFR expression, which then limits proliferation [60]. Furthermore, the BMP gradient along the crypt-villus axis directly results in different hormone excretion profiles of entero-endocrine cells [61]. All of these signaling pathways involved in the murine intestinal epithelium serve as a great model for human intestinal biology. However, the colonic stem cell dynamics in mice are much less defined and the murine colonic epithelium is much less susceptible to malignant transformation in many models. This is important because human CRC mostly arises in the colonic tract and these pathways might elicit different effects on human colonic epithelial cells. This should be anticipated when translating knowledge obtained in the murine small intestine, to the human situation.

#### Colonic stem cells

The murine colonic epithelium shows a similar crypt-structured pattern as compared to the small intestine but lacks villi. The colonic crypt is also populated with stem cells in the bottom that produce specialized cells that cover the crypt wall. However, the cellular composition differs from the small intestine as Paneth cells, the ‘+ 4’ population and *Bmi1*^+^ cells are absent. However, other crypt bottom cells (e.g. cKIT^+^ and Reg4^+^ cells) intermingling with the Lgr5^+^ population are present and express growth factors reminiscent to Paneth cells in the small intestine [62, 63]. Also, Wnt signals derived from the mesenchymal cell population surrounding the colonic crypts are critical for stem cell renewal and tissue maintenance [64]. Colonic stem cells have also been identified as Lgr5^+^ and EphrB2^high^ [14, 65, 66]. Additionally, cell cycle differences have been found among the colonic stem cell population, of which high *Notch* and *Lrig1* expression mark the slow cycling population [22, 67].

Clearly, applying transgenic lineage tracing techniques in humans is not feasible. However, different lineage tracing techniques based on neutral somatic mutations have been successfully applied to study stem cell dynamics in the adult colon [26, 68]. These studies show an estimated number of functional colonic stem cells that each contribute in a stochastic fashion to spawning new clonal lineages, ranging between five to six or five to ten active stem cells [26, 68]. Importantly, human stem cells have a significant slower niche fixation rate compared to their murine intestinal counterparts. On average one colonic stem cell is predicted to be replaced every year within a crypt in contrast to the murine colon where the replacement rate is much higher, namely every three days [30, 68]. Hence, while general concepts of stem cells dynamics are conserved between mice and humans, the rates can be highly different.

### Tumor initiation in the intestine

#### Cell-of-origin

**Fig. 2:**
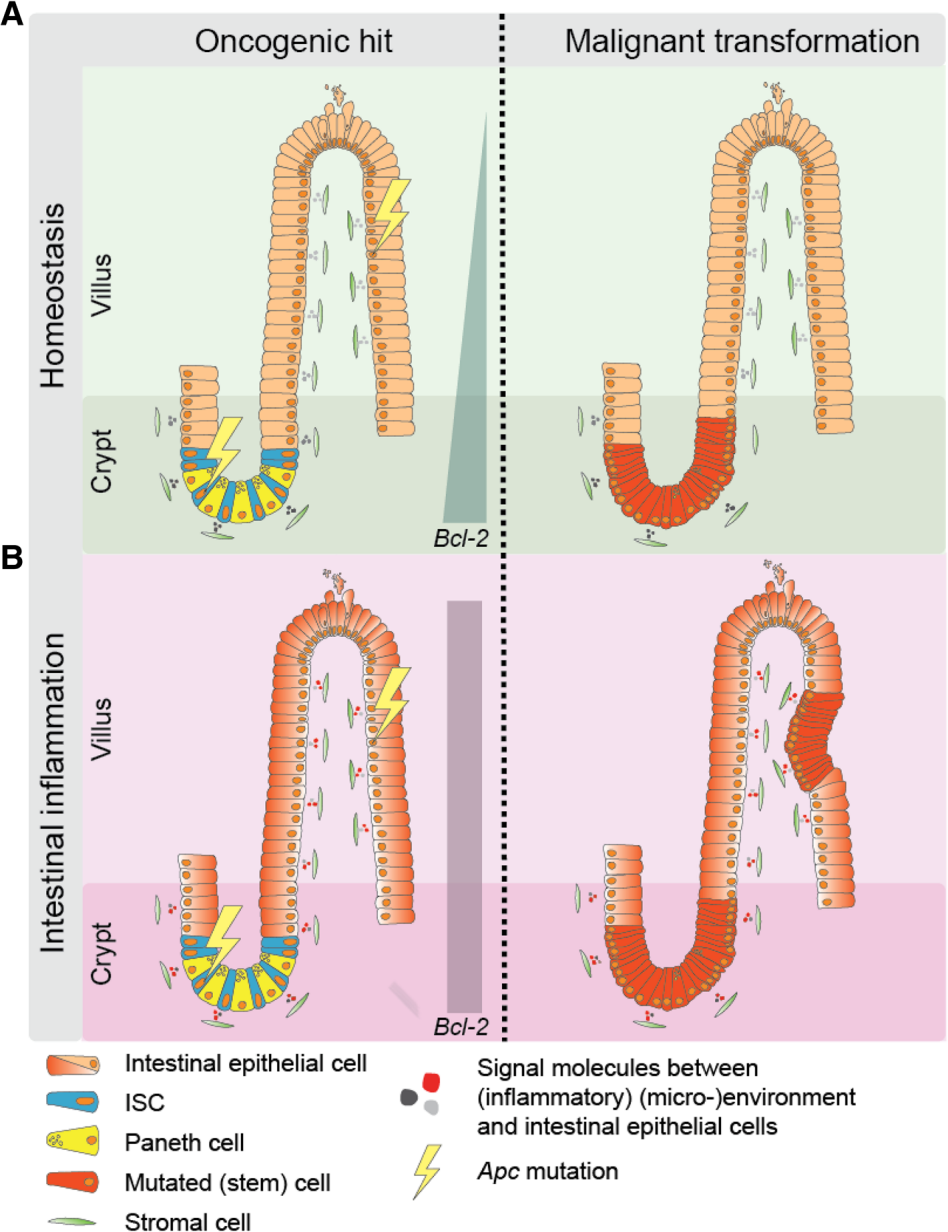
Intestinal cell plasticity dynamics in malignant transformation. (**a**) ISCs have the ability to effectively initiate adenoma formation when these cells acquire *Apc* mutations. On the other hand, differentiated intestinal epithelial cells do rarely undergo malignant transformation upon obtaining an oncogenic hit. (**b**) However, in an inflammatory environment differentiated cells acquire similar transformation potential. Different factors underlie the differences in transformation capacity of ISCs versus differentiated cells. First of all, the topological position of differentiated cells prevents them in homeostasis from generating long-lived clonal lineages. Secondly, the ISC niche endows ISCs with the potential to endure the stressors that result from acquiring an oncogenic mutation [74]. Similarly, in a colitis environment the differentiated cell compartment is also installed with anti-apoptotic capacities through activation of the nuclear factor-κB (Nf-κB) pathway [73]. The anti-apoptotic protein BLC-2 is one of the identified mediators that facilitates this oncogenic transformation. Indeed, inhibition of BCL-2, either genetically or pharmacologically, reduced adenoma burden in mice [74]

Generally it is assumed that the ‘cell-of-origin’ for most cancers is a tissue-specific stem cell [69]. Evidently, their long-term clonogenic potential required for tissue sustenance makes stem cells ideal candidates to accumulate DNA alterations and initiate cancer. Also, in case of the intestinal epithelium, the stem cell compartment is life-long maintained, in contrast to the differentiated cells that are shed into the lumen within a week. Evidently, this limits their potential to clonally expand. However, morphologic analysis of human adenomas showed evidence that in some cases, the intestinal cells higher up in the crypts are responsible for adenoma initiation and not the stem cells in the crypt base. This has been posted as the ‘top-down’ model for adenoma initiation [70]. The large majority of the CRCs harbor a mutation in the adenomatous polyposis coli (*APC*) gene, and this gene is identified as one of the initial oncogenic events in CRC [3]. The APC protein is a key member of the β-catenin degradation complex [71]. Mutations within APC result in ineffective targeting of β-catenin for degradation and causes a constitutively active Wnt pathway which results in an expanding ISC compartment followed by adenoma formation [72]. In genetic mouse models adenomas only appeared when this mutation was specifically introduced in ISCs, for example in in Lgr5^+^, *Bmi1*^+^ or *Prom1*^+^ cells [11, 17, 23], while *Apc* mutations targeted to the differentiated cells only resulted in indolent cystic structures [11] (Fig. 2a). In contrast, full adenomatous outgrowth was also observed upon combined activation of constitutive active Wnt and the nuclear factor-κB (Nf-κB) pathway in the differentiated compartment (Fig. 2b) [73]. As we previously showed, one of the key mediators that allows for ISC transformation is the anti-apoptotic protein BCL-2 which is both highly expressed in Lgr5^+^ CBCs and a target gene of the Nf-κB pathway [74]. Moreover, given the ability of intestinal epithelial cells to undergo extensive plasticity during tissue damage and regeneration, it seems likely that inflammatory signals from the environment install differentiated cells with a similar oncogenic potential as the ISC cells. Another example is the post-mitotically differentiated Tuft cell population which in homeostasis do not contribute to tissue renewal, but in case of intestinal injury displays ISC activity and also intestinal polyp forming capacity only in a colitis setting [75, 76]. We speculate that functional cellular transitions occur under influence of extrinsic factors and a major role seems to be reserved for the cellular (micro-)environment. Another observation supporting this hypothesis, is the increased risk for CRC development in patients with chronic colitis [77, 78] and the reduced risk of colorectal adenoma development upon anti-inflammatory drug treatment, such as celecoxib and aspirin [79, 80]. Reduction of an inflammatory phenotype via COX-2 inhibition led to a decrease in polyp burden [81] and reduces the risk for the development of colorectal cancer [82]. Therefore, (micro-)environmental factors that enhance inflammatory pathways, e.g. Nf-κB pathway activation, seem to underlie the risk of CRC development. Potentially by expanding the pool of cells amendable for malignant transformation.

#### Niche fixation of mutated cells

Mutations that are involved in the malignant transformation of intestinal epithelial cells have been well-defined [3]. For CRC, in many cases this involves a mutation in the tumor-suppressor genes *APC* and *TP53*, and the oncogene *KRAS* [3]. Recently, the quantitative dynamics of these mutations and the impact on the clonal behavior of ISCs have been investigated by us and others [83, 84]. As mentioned above, the fast-cycling CBCs are most susceptible for initiating clonal lineages that carry a specific mutation. In homeostasis, ongoing stochastic competition takes place among the 5–7 functional ISCs [27, 28]. These dynamics follow the ‘neutral drift’ model, indicating the random replacement behavior of ISCs in the crypt bottom. However, when an ISC acquires an oncogenic mutation, for example in *Apc* or *Kras*, a bias ensues in favor of the mutant cells (biased-drift) [83, 84]. For example, the probability of a *Kras*^G12D^ mutated cell to replace its neighbor ISCs and finally become clonal within a crypt is respectively 60–70% in comparison with 12,5–20% for non-mutated ISCs [83, 84]. Importantly, although the mutated ISC gains a higher probability of niche fixation, these cells are still subjected to undergo replacement by normal ISCs. Interestingly, *Trp53* mutations only present with a superior niche fixation rate in case of colitis, which again underscores the importance of extrinsic factors in malignant transformation of intestinal cells [83].

The morphological tissue architecture of the intestine prevents rapid spreading of mutated cells as each of these crypts is a dynamic cellular niche on its own without any exchange of cells in between crypts. However, the number of crypts is not stable due to two processes called fission and fusion, meaning respectively bifurcating and colliding crypts [85]. These counteracting processes compensate for each other and are both in man and mice an infrequent event, unless tissue damage occurs [85–89]. Interestingly, a much higher fission rate is observed in *Kras* mutated crypts [84]. This is also illustrated by the notion that multiple *KRAS* mutated neighboring crypts can be found surrounding a CRC, suggesting that within a *field* of *KRAS* mutant crypts one crypt has undergone further transformation [90]. Therefore, crypt fission seems to be an important mechanism for malignant transformation and progression in the intestine, involving a process referred to as field cancerization. In a clinical setting, it would be relevant to therapeutically prevent the process of field cancerization, as it would significantly decrease the risk of CRC development by simply keeping the numbers of mutated crypts as low as possible.

### Clonal expansion in the intestine

#### Adenoma formation and growth

Once an adenoma is formed upon expansion of mutated crypts and at least 1 cm in size, there is a ~ 25% risk of this newly formed adenoma to undergo malignant transformation towards an invasive carcinoma in the following two decades [91]. Mechanisms that underlie this progression are nearly impossible to capture in humans. Unfortunately, there is also a lack of tumor mouse models that mimic invasive growth. Nevertheless, several groups successfully investigated the stem cell compartment in these benign tumors in both mice and man [30, 92, 93]. First of all, the morphology of adenomas containing glandular structures closely resembles the healthy crypt-structured intestine and these also contain a range of different cell types [92]. Upon adenoma initiation in Lgr5^+^ cells, lineage tracing was performed by so-called ‘re-tracing’ of the Lgr5^+^ population in established adenomas. This showed that also in adenomas the Lgr5^+^ cell population in the bottom of glands, display a similar repopulating potential as their normal counterparts within the glandular structures [92]. Similarly, clonal tracing from the rare doublecortin-like kinase 1 (*Dclk1*) positive cell population in the *Apc*^min^ mouse model showed the clonogenic properties of these cells and ablation of the *Dclk1*^+^ cell population results in adenoma volume reduction [76]. We contributed to further quantitative insight into the stem cell dynamics within adenomatous tissue using a marker independent clonal tracing strategy [30]. This method revealed that in adenomatous crypts ~ 9 functional stem cells are present per hundreds of cells within each gland. This is in contrast with the percentage of Lgr5^+^ cells that is found within the adenomas, approximately ~ 20% of the total population (~ 400 cells per gland). Therefore, it seems unlikely that each Lgr5^+^ cell exhibits similar stem cell activity [92]. Similarly, in case of human adenomas, multi-lineage differentiation was demonstrated within glandular structures, which suggests the existence of multi-potent stem cells [93]. Here, clonal tracing was performed by exploiting the random occurrence of stable non-oncogenic mutations in the mitochondrial genome that can be visualized by immunohistochemistry within individual adenomatous cells [93]. Methylation patterns of different clonal patches were very heterogeneous which indicates that already at early-stage tumorigenesis intra-adenoma (epigenetic) clonal diversity arises [93]. However, the underlying dynamics and effects on clonal behavior during malignant transformation have remained largely unresolved. Furthermore, gland fusion events are limited in adenomas and gland fission is assumed to be an important mechanism by which adenomas increase in size [94, 95]. Hypothetically, targeting crypt or adenoma gland fission events would be an attractive method to prevent the process of field cancerization or halt adenoma growth. However, further mechanistic insights would be necessary in order to develop these therapies.

### Intestinal cancer stem cells

#### Intestinal stem cell plasticity and (micro-)environmental influences

In analogy with intestinal epithelial turn-over, for many years it is believed that CRC growth and progression are fueled by a dedicated cancer cell population that possesses self-renewal and multi-potency potential, and these cells are referred to as cancer stem cells (CSCs) [96, 97]. The presence of a cellular hierarchy explains the cellular heterogeneity, with respect to the differentiation grade, that is found within CRC [6, 98]. This paradigm has been around for many decades and besides therapy failure may also explain phenomena such as tumor dormancy and metastasis. The normal intestinal epithelium displays great regeneration capacity upon injury due to the potential of epithelial cells to easily switch between differentiation states [31–35, 39]. In addition to specialized epithelial cells as Paneth and cKIT^+^ cells, it is well established that also the stromal compartment constitutes for crucial signals that are needed to equip cells with ISC functionality [49, 64]. Moreover, an inflammatory environment is another facilitating component that installs ISC functionality and thereby enhances the malignant transformation capacity of differentiated cells [73, 74]. Similarly, in human CRC, different signals directly derived from the tumor (micro-)environment have been found to induct a CSC phenotype and CSC functionality [5, 6, 99]. We would argue that plasticity of CRC cells is likely to be more pronounced as compared to normal intestinal epithelial cells although of course this is difficult to directly compare. This plasticity is exemplified in a mouse model that mimics human CRC growth and also a human CRC xenograft model [4, 100]. Here, therapeutic ablation of the tumor-specific Lgr5^+^ cells in xenografts initially leads to impaired tumor growth [4, 100]. However, shortly after discontinuation of Lgr5^+^ cell depletion therapy, tumor growth resumes at similar growth rates as untreated control tumors [4, 100]. Specifically, Wnt-activating factors that are secreted by the stromal myofibroblast cell compartment include factors like hepatocyte growth factor (HGF) and Osteopontin, have demonstrated to elicit the CSC phenotype or activity [5, 6]. In addition, TGFβ has similar effects but also elicits a migratory and pro-metastatic phenotype in cancer cells, either directly or via the cancer associated stromal cells [101–103]. Importantly, dedifferentiation of non-CSCs to CSCs is predicted to greatly hamper effective responses to specific CSC targeted therapies [96]. Another complicating factor is the activating effect on tumor-associated stromal cells upon cytotoxic treatment. These cells show an increased secretion of specific chemokines and cytokines, e.g. interleukin-17A, that is able to sustain the CSC compartment [104]. Altogether, these studies suggest that specifically targeting the CSCs within CRC will likely not be sufficient. The CSC state is not a fixed entity due to intrinsic features, but rather highly dynamic and driven by environmental cues. In parallel, blocking of the (micro-)environmental signals that are derived from the tumor niche seems crucial in order to avoid replenishing of the CSC pool.

#### Identification of the intestinal cancer stem cell

This dynamic nature of CSCs complicates identification of the CSC pool in established CRC. Similarly for the normal intestine, previously distinct ISC states have been summarized in a comprehensive manner by four terms: the ISC phenotype, activity, potential and functionality (for description of these terms see Table 1, 38). Analogously, there is evidence that the highly dynamic nature of the intestinal epithelial cell compartment is mirrored in CRC and therefore identifying one defined CSC population that is unchangeably present in all circumstances has proven to be complex [6]. Initial efforts to identify the CSC population, mostly based on identification of the CSC phenotype and activity, started over a decade ago and has provided tremendous insights into cancer biology [97]. CSCs were identified based on differences in cell-surface marker expression and this essentially reflected the CSC phenotype and activity. This method originated from the field of hematological malignancies [105]. In these diseases it was shown that a subpopulation of leukemic cells that express cell-surface markers associated with immature cell types, was able to transmit leukemia upon injection into immune-compromised mice [106]. Hence, it was suggested that this method was also useful for distinguishing between the CSC and non-clonogenic differentiated/progenitor cell population in solid malignancies [97, 107, 108]. For long, the gold standard assay to test CSC activity in solid cancers was to study the tumor initiating capacity of cancer cells upon single cell sorting for these markers and then determining tumor outgrowth following subcutaneous or orthotopic injection of these cells in mice. In case of CRC, multiple markers have been identified that were designated to reveal the CSC identity: CD133^+^, EpCAM^high^/CD44^+^/CD166^+^, ALDH^+^, EphB2^high^, and Lgr5^+^ [65, 109–115]. Furthermore, additional markers have been described that are associated with specific CSC subsets characterized by distinct features. For example, colorectal CSCs with a marked potential to form distant metastasis are identified by CD26 and CD44v6 surface expression [99, 116] Further evidence for the unique role of CSCs in the metastatic process comes from the finding that cells expressing these markers (CD26 and CD44v6) can be isolated from the blood of CRC patients as circulating tumor cells (CTCs), and these cells display the ability to form cancers [117].

Unfortunately, the straight-forward idea of discriminating between CSCs and non-CSCs based on differential marker expression and clonogenic potential in xenotransplantation assays has proven to be opportunistic. Certain caveats are present, such as that using marker expression for CSC identification in many cases involves the use of proteins that directly facilitate grafting, e.g. CD44 [97, 118]. Secondly, similar to ISCs, the CSC state seems to be highly dynamic and partly installed by (micro-)environmental signals rather than a fate caused by intrinsic features [6]. Thirdly, the heterogeneous nature of malignancies is reflected in widespread heterogeneity between individual cancers of the same type, and even between clones, when CSC markers are considered [110, 119–121]. Furthermore, CSC marker expression is dynamic and therefore varies in time [122]. Critically, the xenotransplantation assays described above, solely capture CSC potential in an artificial manner as it requires disruption of tumor tissue. However, the process of tumor growth evidently relies on which cancer cells display clonogenic capacity within tumor tissue, so called CSC functionality, which has not been examined by using the transplantation assays. In the next section new techniques, i.e. (genetic) lineage tracing, are discussed that study CSC functionality in situ*,* which will ensure investigation of the dynamics of CSCs in tumor growth. This is important for further understanding of CRC in minimal residual disease, under therapeutic pressure and upon metastasizing to distant organs.

#### Cancer stem cell functionality

**Fig. 3:**
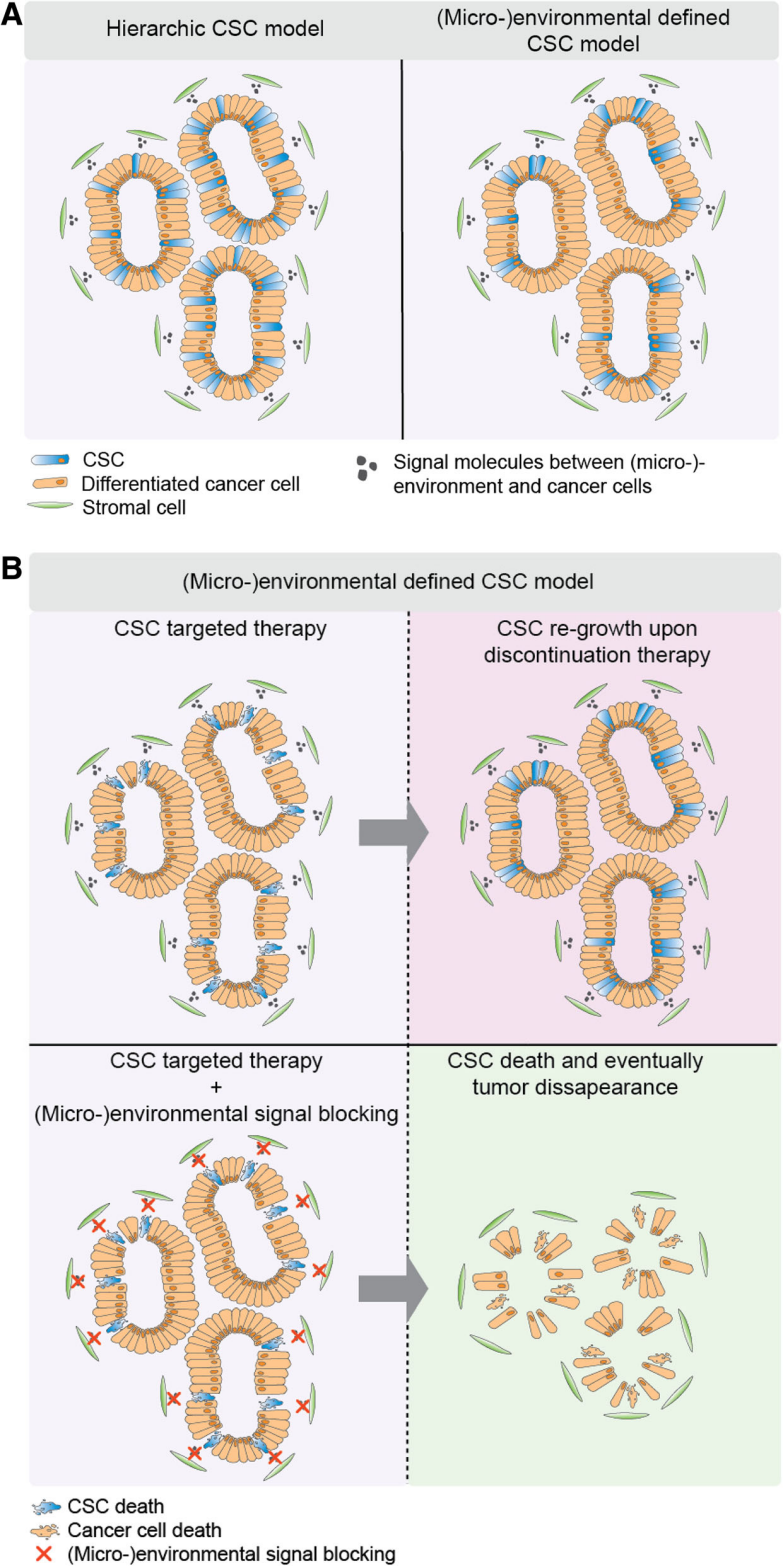
Cancer stem cell dynamics in colorectal cancer growth and therapy. (**a**) The strictly hierarchical cancer stem cell (CSC) model postulates that the CSC state is a fixed entity and CSCs are intrinsically equipped with self-renewal potential and multi-potency. On the opposite, the (micro-)environmental defined CSC model states that signal molecules derived from the stromal compartment install CRC cells with CSC potential, such as self-renewal and multi-lineage differentiation capacity. (**b**) The top panel predicts that eradicating CSCs by blocking important stem cell signaling pathways, e.g. Wnt signaling, is not sufficient to halt tumor growth. Once treatment is discontinued (top left panel) specific CSC-installing signals from the niche will provide CRC cells with CSC potential and these CSCs will again drive tumor growth. The bottom panel shows a situation in which both (micro-)environmental signals as CSC-specific pathways are blocked resulting in potentially effective tumor control

Strategies that involve (genetic) lineage tracing have been a widely used tool to study (stem) cell and clonal dynamics in different murine organs and their tissue-specific malignant counterparts [123]. However, the use of lineage tracing in human tissues and xenografts has been limited, as has the use of quantitative models of CSC-driven cancer growth. Conceptually dynamics of CSC populations are radically different than those of stem cells sustaining normal tissue homeostasis, as stem cells in cancer are an expanding population whilst in healthy organs the stem cell number remains constant. This notion has important implications for the models employed to describe the dynamics of the stem cell pool in cancers. Recently, we used a direct marker-free lineage tracing approach to investigate CSC functionality during short-term CRC outgrowth in an unbiased fashion [5]. Here, a clear heterogeneity in growth dynamics of the cancer cell pool was demonstrated within different tumor regions, e.g. cells located near the border or closer to the center. Strikingly, clonogenic outgrowth occurs mainly at the tumor border as opposed to the tumor center. Predictions of an accompanying mathematical model shows that the observed CSC dynamics can be attributed to (micro-)environmental regulation instead of cell-intrinsic features, thereby disregarding the strict hierarchical CSC theory (Fig. 3a). Additionally, in this model no correlation was found between CSC functionality and the CSC phenotype, as the presence of Lgr5^+^ cells was equally distributed throughout the whole tumor. In comparison, two other studies demonstrated that the Lgr5^+^ population represents the functional CSC pool compared to the more differentiated cell types [4, 100, 109]. However, spatiotemporal dynamics of these Lgr5^+^ cells have not been specified. Another recent study highly supports the surface growth-driven model of CRC [124]. In this study neutral and stable multi-color labeling of CRC cells was employed to investigate the clonal outgrowth during the process of tumor growth. Strikingly, CRCs clearly show marked clonal outgrowth at the tumor edge in the whole process of CRC expansion and progression. These data also serve as an explanation for earlier observations from genetic barcoding studies [125–127]: Interestingly, these studies showed that upon clonal tracing in transplantation assays, different clones seem to either disappear or re-appear in serial transplants, which is an observation that often is attributed to the intrinsic CSC potential of cells. However, in agreement with the environment directed surface growth model, clones on the tumor border exhibit the greatest clonogenic potential due to their privileged location close to (micro-)environmental stimuli. However, when upon re-transplantation cells from smaller clones that have resided in non-privileged tumor sites extensively contribute to tumor growth, this does not reflect an intrinsic feature but simply more optimal environmental support. Evidently, studying CSCs and clonal dynamics in primary CRC in humans requires different approaches, as it is unethical to systematically observe tumor growth in patients. Techniques that infer clonal dynamics by taking advantage of neutral differences in the genomic composition of cells have been developed. For example, determining modifications in the metastable methylation pattern of CpG-rich genomic regions has proven to be a useful tool in CRC samples for this purpose as a measurement for the CSC fraction [128, 129]. CSC estimations derived from these two studies were fairly dissimilar; one group inferred a functional CSC fraction of 1% from total population [129] whereas another group also speculated on a much higher incidence of functional CSCs [128]. Therefore, future studies that study the functional CSC compartment in human CRC would largely benefit for improved methods such as greater in-depth DNA sequencing or new lineage tracing tools based on neutral and stochastic genomic alterations [68].

### Cancer stem cells in therapy

The frequent occurrence of therapy resistance remains one of the major clinical challenges for anti-CRC treatment. Multiple mechanisms underlie this therapy resistance for systemic therapies. Broadly, two main mechanisms have been described: genetic, either innate or acquired, and non-genetic mediated resistance [130]. CSCs are often held responsible for therapy resistance and indeed could provide an explanation for the observed non-genetic resistance patterns. In addition, CSCs provide an attractive explanation for the phenomenon of minimal residual disease in which seemingly effective therapy is followed by a remission due to the outgrowth of few surviving cancer cells [96]. Indeed, it was demonstrated that colorectal CSCs present with an increased resistance to conventional cytostatic agents [104, 131–134]. However, one major limitation in these pre-clinical studies is the phenotypic outcome measurements as interpretation for successful targeting of anti-tumor growth, e.g. investigation of reduced marker expression or Wnt signaling activity. Most likely this does not reflect the in situ CRC resistance. Nor does it provide information on the extent of therapy resistance of the clonogenic core of the cancer, i.e. the functional stem cell pool. Therefore, dedicated techniques that study the functional CSC compartment in space and time should be used to study CSC behavior upon therapeutic interventions. Examples of these methods include serial passaging of xenograft material, assessment of metastatic capacity and the ability to drive regrowth of cancers after therapy cessation. In addition, specific measurement of clonogenic potential in situ of treated cells using lineage tracing strategies is feasible [5].

Various different characteristics are designated to CSCs that are thought to be important for their resistant phenotype. One example is the predicted dependence of CSCs on highly conserved signaling transduction pathways that are also involved in normal stem cell biology [6, 135]. These pathways include for example Wnt, Notch and Hedgehog (HH). Therefore, one approach as anti-CSC strategy is to inhibit these pathways. For example, in case of CRC compounds that directly inhibit the Wnt pathway or target the Wnt^high^ cells have been generated. In xenograft studies it was shown that specifically targeting the Lgr5^+^ cells through antibody-drug conjugated therapy, or directly genetically, indeed inhibits tumor growth without affecting intestinal epithelium homeostasis [4, 136, 137]. Also, other upstream Wnt pathway inhibiting agents have been described to halt tumor growth in preclinical models [138, 139]. Several clinical trials are currently running to test the effect of CSC interference on tumor growth. For CRC, these inhibitors include the upstream Wnt-signaling targets, e.g. PORCN and anti-RSPO3 [140]. Critically, the methods used to determine treatment responses are often criticized as these are solely based on the outcome of surrogate parameters, e.g. radiological tumor response in early phase trials. Yet, similar to preclinical studies, it would be crucial to measure the therapeutic effects on specifically the (functional) CSC compartment.

In addition, potentially multiple other factors complicate the efficacy of anti-CSC treatment in patients. For example, differential therapy responses might occur depending on the location of the CSCs, either in (loco-)regional or hematogenous metastasized CRC. Namely, CSC activity and functionality might differ depending on which environmental stimuli these cells receive from their tumor niche [4]. Interestingly, the Wnt^high^ cell population is found responsible for metastasis to distant organ sites such as the liver [4]. This indicates that targeting the Wnt pathway could be beneficial for preventing metastasis. Importantly, most (pre-)clinical trials are performed in patients that already have metastatic disease. Furthermore, it is conceivable that inhibiting the Wnt signaling cascade is unpromising as the tumor niche and its crosstalk with tumor cells mediates dedifferentiation of non-CSCs. This implies that replenishment of the CSC pool still occurs when CSCs are targeted but not (micro-)environmental stimulated dedifferentiation of other CRC cells (Fig. 3b). Ideally, an approach with combined treatment of inhibiting tumor-niche signals installing the CSC state and a direct anti-CSC target would be essential (Fig. 3b). An example of targeting the Wnt agonizing stimuli from the (micro-)environment would be to block the MET receptor, preventing activation by myofibroblast-derived HGF [141, 142]. Interestingly, one study found that monotherapy with targeting the Wnt^high^ CRC cells in liver metastasis was sufficient to prevent re-growth of tumors [4]. This indicates that different organs provide for distinct tumor niches which impacts on the extent of CRC cell plasticity. Another phenomenon that might hamper effective anti-CRC treatment is the stochastic phenotypic state switching events of tumor cells. For breast cancer it was found that in vitro the cancer cell population was stably displaying a constant phenotypic equilibrium, even upon isolation and expansion of distinct subpopulations [143]. This suggests that cancer cells are subjected to stochastic (de-)differentiation, even without the interference of (micro-)environmental stimuli. This mechanism of stochastic transitioning between differentiation states of tumor cells, in addition to (micro-)environmental mediated CSC plasticity, might be a major contributor to therapy resistance, which at present is complicating the efficacy of anti-CRC therapies.

## Conclusions

Clearly, intestinal homeostasis is a much better understood process than the dynamics that underlie CRC formation and growth. However, also for the normal intestinal epithelium critical unsolved issues remain to be answered. For example, it is still unknown to which degree intestinal cellular plasticity takes place and whether all intestinal epithelial cells are able to undergo such events. Similar to the normal intestinal epithelium, for CRC it has been recognized that a cellular hierarchy is present [97]. Also, it is apparent that CRC progression, which relies on CSC activity, does not simply depend on the mutational profile of tumor cells within different clones or tumors. Particularly, it is proposed that CSC activity is coordinated by the niche and possibly stochastic events instead of intrinsic regulatory mechanisms [5, 124, 143]. Evidently, CRC cells are involved in a dynamic interplay with their niche, and interact through the modification of multiple signaling pathways that are yet partly unknown. Identification of the key players that provoke CSC activity in CRC cells will be crucial. In addition, we and others in the field raise critical concerns about the predicted effectiveness of strategies to cure CRC that solely target intrinsic stem cell features [140, 144]. Furthermore, (micro-)environmental regulation of tumor cells might also depend on the organ specific environment [4], which potentially will complicate the development of suitable therapies. Another hurdle is the potential heterogeneity of the stromal compartment among different CRC subtypes, which would require different approaches for abrogating stroma-tumor interactions [9]. Future studies should be directed towards gaining a better understanding of CSCs behavior in human tumor growth and upon therapy responses, with the emphasis on studying CSC dynamics in their native environment. These insights will be crucial for developing new strategies to more effectively treat this disease.

## Abbreviations

APC: Adenomatous polyposis coli
Bmi1: B lymphoma Mo-MLV insertion region 1 homolog
BMP: Bone morphogenetic protein
CRC: Colorectal cancer
CSC: Cancer stem cell
Dclk1: Doublecortin-like kinase 1
Dll1: Delta-like 1
Dll4: Delta-like 4
EGF: Epithelial growth factor
HGF: Hepatocyte growth factor
HH: Hedgehog
Hopx: HOP homeobox
ISC: Intestinal stem cell
Lgr5: Leucine-rich-repeat-containing G-protein-coupled receptor 5
LRC: Label-retaining cell
Lrig1: Leucine Rich Repeats And Immunoglobulin Like Domains 1
mTert: Mouse telomerase reverse transcriptase
Nf-κB: nuclear factor-κB
Prom1: Prominin 1
Smoc2: SPARC related modular calcium binding 2
Sox9: SRY-box 9
TGF-α: Transforming growth factor-α
